# Study of Novel Geopolymer Concrete Prepared with Slate Stone Cutting Sludge, Chamotte, Steel Slag and Activated with Olive Stone Bottom Ash

**DOI:** 10.3390/ma18091974

**Published:** 2025-04-26

**Authors:** Raul Carrillo Beltran, Elena Picazo Camilo, Griselda Perea Toledo, Francisco Antonio Corpas Iglesias

**Affiliations:** Research Group TEP 222 “Materials and Mining Engineering”, Higher Polytechnic School of Linares, University of Jaen, 23700 Linares, Jaen, Spain; epicazo@ujaen.es (E.P.C.); gept0001@red.ujaen.es (G.P.T.); facorpas@ujaen.es (F.A.C.I.)

**Keywords:** geopolymer, slate stone cutting sludge, chamotte, olive biomass bottom ash, steel slag, geopolymer concrete

## Abstract

The expansion of the construction sector has contributed to the depletion of raw materials and an increased demand for resources; therefore, sustainable approaches are required to satisfy the construction demand. The present study explores the development of geopolymers by utilizing industrial by-products from mining, ceramics, olive oil production, and steel manufacturing. Specifically, slate stone cutting sludge (SSCS) and chamotte (CH) are used as aluminosilicate precursors, with olive biomass bottom ash (OSBA) acting as an alkaline activator, along with sodium silicate, and steel granulated slag (SGS) incorporated as an aggregate. Novel geopolymers were prepared with consistent proportions of SSCS and OSBA while varying the CH content from 10 to 2 wt.%. The SGS proportion was adjusted from 35 to 50 wt.%, and different Na_2_SiO_3_/OSBA ratios (0.35, 0.31, 0.19, and 0.08) were examined. To identify the optimal mix, a series of physical and mechanical tests was conducted, complemented by FTIR and SEM analysis to evaluate the chemical and microstructural changes. The best-performing formulation achieved a compressive strength of 42.8 MPa after 28 days of curing. FTIR analysis identified quartz and carbonate phases, suggesting that quartz did not fully dissolve and that carbonates formed during the heating process. SEM examination of the optimal mixture indicated that the incorporation of SGS (up to 45 wt.%) facilitated the creation of a compact, low-porosity structure. EDX results revealed the presence of Ca-, Na-, Si-, Al-, and K-enriched phases, supporting the formation of (N, C)-A-S-H gel networks. These results demonstrate the potential of utilizing SSCS, CH, OSBA, and SGS to create geopolymer concretes, showcasing the viability of using industrial by-products as eco-friendly substitutes for traditional construction materials.

**1.Introduction**

The need to supply raw materials to meet the growing demand in the construction and building sector, which is forecast to exhibit a 57% increase in volume globally by 2030 [1,2], is causing certain industries such as mining and steel manufacturing to have a strong influence on solid waste worldwide [3], as well as CO_2_ emissions and energy consumption [4]. The 2022 statistics report on waste generated by EU economic activities indicated that the construction sector accounts for 38% of the total estimated volume, primarily due to mining and quarrying (23%), steel manufacturing (10%), and energy production (4%), among others [4].

With the aim of mitigating the negative impacts associated with the use of ordinary Portland cement-based construction materials, material science researchers are boosting the study of construction materials that reduce the amount of OPC [5,6,7,8,9] or do not use it in their formulations [10,11,12,13,14], leading to a more sustainable material. Geopolymers can be a potential example of an alternative material and have received great interest in the scientific community due to their environmental advantages in comparison to ordinary Portland cement (hereinafter referred to as OPC) materials. However, these advantages are subject to the type of geopolymer mixes, and a rigorous life cycle analysis is needed to quantify the impacts [15,16].

Geopolymers are considered environmentally beneficial due to their inclusion of raw materials sourced from the recycling of various by-products rich in silica and reactive alumina [17]. These by-products can be activated either by combining them with conventional alkaline activator solutions [18] (such as potassium or sodium hydroxide or potassium and/or sodium silicate) or by using alternative activation methods [19,20,21].

Previous studies have explored the development of geopolymers through the valorization of various agricultural, industrial, mining, and human-made wastes, such as biomass ash [22,23,24], slag [25,26,27,28], chamotte and red ceramics [29,30,31], cutting sludge and mining tailings [32,33,34], construction and demolition waste [35], and glass from urban waste [36]. Given the diverse sources of aluminosilicates, researchers have demonstrated that the final properties and characteristics of geopolymers—such as low thermal conductivity [37], fire resistance [38], acid resistance [39], and compressive strength [17]—are closely linked to the types of raw materials used in the geopolymerization process.

Given the factors mentioned above, geopolymerization technology may be chosen as a sustainable approach for managing the uncontrolled landfill of by-products from different sectors, such as mining, ceramics, olive cultivation, and steel production.

In Spain, the slate stone mining industry, the ceramic industry, olive oil production, and steel manufacturing all contribute large quantities of waste. Slate mining in northern Spain produces tailings that have accumulated due to high international demand for slate products. The ceramic industry generates a significant amount of defective fired clay products, which can be repurposed for road construction or as aggregates. Olive oil production, particularly in Andalusia, results in the accumulation of olive stone bottom ash, which has limited reuse potential. Steel slag, a by-product of steel production, often ends up in landfills, where it poses environmental risks like heavy metal leaching.

Therefore, this research is focused on the study of the potential valorization of cutting sludges from slate stone mining, chamotte from ceramic factories, slag from steel manufacturing, and olive stone bottom ash from power generation. This study aims to analyze the feasibility of the combination of these by-products for the development of novel geopolymers with similar features to OPC materials with aggregates.

The rationale for choosing these by-products is that previous scientific studies [40] have demonstrated that slate stone contains a high proportion of alumina and silica, making slate stone cutting sludge (hereinafter referred to as SSCS) a promising source of aluminosilicates for geopolymers. Similarly, chamotte (hereinafter referred to as CH) has been investigated as an ideal precursor for geopolymer synthesis due to its aluminosilicate composition [12] and for its pozzolanic activity [41]. With regard to the alkali activator solution, olive biomass bottom ash (hereinafter referred to as OSBA), as by-product of olive oil production created through the calcination of olive stones, contains a high potassium content and therefore can be used in the production of an alkali activator solution in combination with sodium silicate. Finally, steel slag plays a role as an aggregate in the geopolymer, offering a sustainable approach for recycling. Steel slag has a pH value between 8 and 10, which makes it alkaline. However, a pH higher than 12 can be observed due to a high amount of free calcium oxide compounds in the slag [42]. The fact that the steel slag has a relevant alkaline pH makes it possible to consider as a potential sustainable aggregate to be combined with the aforementioned mining, ceramic, and biomass by-products.

Based on the potentials of the mentioned by-products, SSCS and CH were used as sources of aluminosilicates, while OSBA, sodium silicate, and distilled water were used to prepare the alkali activation solution. Steel granulated slag (hereinafter referred to as SGS) was selected as an alternative aggregate for the geopolymer binder.

During this research study, the chemical and physical characterization of by-products and the physical and mechanical characterization of novel geopolymers were carried out.

This research study was concluded by demonstrating the feasibility of geopolymer concrete made with SSCS, CH, OSBA, and SGS (20% SSCS, 8% OSBA, 4% CH, Na_2_SiO_3_/OSBA ratio of 0.19), achieving a compressive strength of 42.8 MPa after 28 days of curing.

## 2. Materials and Methods

This section provides an overview of the by-products utilized as raw materials for geopolymer synthesis, sourced from various industries, including mining, brick manufacturing, olive biomass production, and steel manufacturing. It also outlines the experimental methodology used to evaluate the feasibility of slate stone cutting sludge (SSCS) (Pizarras Matacouta, S.A, Leon, Spain), chamotte (CH) (Baiceram, Bailen (Jaen), Spain), and olive biomass bottom ash (OSBA) (Bioelectrica de Linares, Valoriza Group, Linares (Jaen), Spain), as novel geopolymer precursors. The experimental phase aims to assess the potential of combining raw materials from different origins: (i) slate stone cutting sludge and chamotte served as aluminosilicate precursors; (ii) olive biomass bottom ash, along with sodium silicate, acted as the alkali activation solution; and (iii) steel slag (Siderúrgica Sevillana, Seville, Spain), was used as a recycled aggregate. The control geopolymer was prepared using an alkali activation solution consisting of sodium hydroxide and sodium silicate.

### 2.1. Precursors Materials: Slate Stone Cutting Sludge and Chamotte

Slate stone cutting sludge from La Baña and chamotte from Bailen were selected for use as precursor materials.

La Baña, located in the municipality of Encinedo in the Comarca de La Cabrera, within the Spanish province of León, has a long-standing tradition of slate stone mining and processing, featuring six active slate mining sites. Due to the increased production rates driven by international export demand over the past decade [43], this mining activity has led to a significant generation of waste in the form of tailings and sludges. These by-products resulting from slate stone cutting are a major environmental concern, as they can transport heavy metal particles and potentially contaminate nearby aquifers and land areas. This issue is considered one of the most pressing environmental impacts associated with slate stone mining. In previous laboratory studies using cutting sludges from the La Baña slate mining site, moisture accumulation was assessed by drying the samples in an oven at 105 ± 2 °C for 24 h. Mechanical grinding was not necessary to achieve a smaller particle size, as the natural particle size of the cutting sludges was already suitable for the intended purposes.

The city of Bailén, in the Andalusian Province of Jaén, is a hub of industrial activity, particularly in ceramic brick and tile manufacturing, representing 60% of the ceramic industry in Andalusia. This industry also generates by-products from the rejection of defective or broken ceramic products, which, when accumulated in landfills, pose significant environmental challenges [44]. For the experimental program in this study, chamotte samples were crushed and sieved to obtain a particle size of 250 μm. This process was aimed to increase the reactivity of the precursor material [45] and ensure better homogenization with the slate stone cutting sludges.

### 2.2. Alkali Activator Solution: Olive Biomass Bottom Ash, Sodium Hydroxide (NaOH), and Sodium Silicate (Na_2_SiO_3_)

In this study, two different alkali activator solutions were utilized: (i) for the control geopolymer samples, an activator solution was prepared with distilled water(manufacture, city, abbreviated state (for USA/Canada), country), sodium hydroxide (NaOH)(Panreac Química, Barcelona, Spain), and sodium silicate (Na_2_SiO_3_)(Panreac Química, Barcelona, Spain); (ii) for the novel geopolymer concrete samples, the alkali solution consisted of olive stone biomass ash (OSBA), sodium silicate (Na_2_SiO_3_), and distilled water. The OSBA used in this experiment was a by-product from the combustion of olive stones, leaves, and pruning materials. It was collected from the “Bioelectrica de Linares” waste-to-energy facility operated by the Valoriza Group, located in Linares, Jaén, Andalusia. Previous studies have indicated that an alkali activator containing OSBA, sodium silicate, and water can produce geopolymers with promising mechanical properties, especially when combined with mining and ceramic industry by-products [40].

Given that OSBA is less reactive compared to fly ash [46], primarily due to its larger particle size and the presence of certain impurities, it was first processed through milling to achieve a finer, more consistent particle size. This step is essential, as it enhances the material’s interaction with the activator solution, promoting a more effective geopolymerization process [45].

Furthermore, the OSBA underwent a calcination process at 950 °C for 1 h to increase its amorphous content and enhance the reactivity of its silicon and aluminum components [47,48]. After cooling, the calcined OSBA was sieved to ensure a uniform particle size distribution. This modified alkali activator was then used in the experimental phase of the study.

### 2.3. Steel Granulated Slag Aggregates

The raw material used as an aggregate was granulated steel mill slag (hereinafter referred to as SGS) from the burning of coke, iron, and limestone at a high temperature (1500 °C), collected in Siderúrgica Sevillana (Seville, Spain). SGS is a residual by-product generated during iron production, where the iron ore is reduced by separating the molten metal from the slag [49]. Unlike ground granulated blast furnace slag (hereinafter referred to as GGBS), which is widely used in the manufacture of cement and concrete [50,51], steel mill slag has a higher content of calcium oxide (CaO) and iron oxide (Fe_2_O_3_), which give it mechanical properties that allow it to be used as an aggregate material [52,53]. Its high mechanical strength and durability make it a sustainable and high-performance alternative to conventional aggregates. The GGBS used was subjected to a washing and drying process to eliminate dust particles that would hinder adhesion between the geopolymer mortar and the aggregate material. Once the material was dry, it was sieved into fractions between 22.4 mm and 5 mm to ensure a homogeneous particle size distribution. From an environmental point of view, the use of SGS contributes to reducing the ecological impact of the steel industry by minimizing the accumulation of waste in landfills and promoting a circular economy in the construction sector. Furthermore, its use reduces the extraction of natural aggregates, thus preserving geological resources and reducing the carbon footprint of construction materials.

### 2.4. Chemical and Physical Characterization of SSCS, CH, OSBA, and SGS

The physical characterization phase began with an analysis of the particle sizes of SSCS, CH, OSBA, and SGS to evaluate whether they were appropriate for ensuring the effective activation of the precursor materials during the geopolymerization process. A laser particle size distribution test was conducted in accordance with the UNE-EN 933-1:2012 standard [54] using a Malvern Mastersizer Bruker D8 Venture instrument (Malvern Instruments Ltd., Malvern, UK).

**Figure 1:**
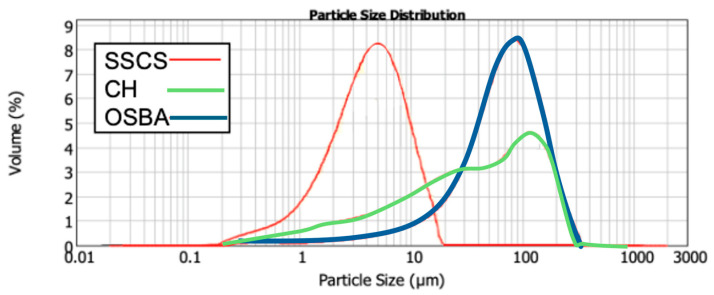
Graphical distribution of SSCS, OSBA, and CH expressed in microns.

**Figure 2:**
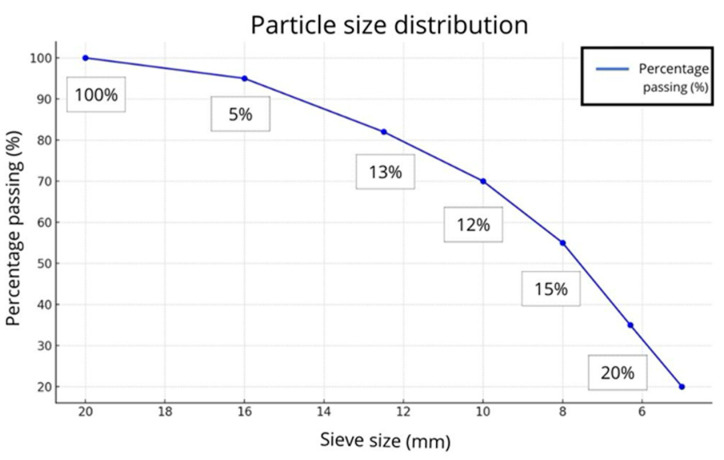
Graphical distribution of SGS particle size expressed in millimeters.

Figure 1 presents the particle size distribution for SSCS, CH, and OSBA, with all particles being smaller than 300 µm. The small particle size of SSCS improves its reactivity in the geopolymerization process due to its large specific surface area (1.88 m^2^/g) [55]. In contrast, CH and OSBA exhibited lower surface areas of 0.794 m^2^/g and 0.321 m^2^/g, respectively. Table 1 shows the particle size distribution at D10, D50, and D90. It is evident that all three raw materials, being fine powders, possess suitable grain sizes that facilitate easy material mixing, which in turn supports the geopolymerization process. Figure 2 displays the graphical distribution of SGS particle size.

The chemical characterization phase began with an analysis of the mineralogical and chemical compositions of the mining, ceramic, and biomass waste materials from power generation. Carbon, hydrogen, nitrogen, and sulfur percentages were determined using a CHNS analyzer (Leco TruSpec Micro Model, St. Joseph, MI, USA). The first step in the chemical characterization involved measuring the nitrogen, carbon, and hydrogen content of the various raw material samples. The results of the elemental analysis for the waste materials used are presented in Table 2.

The results from the elemental analysis tests for slate stone cutting sludge, chamotte, and steel granulated slag, presented in Table 2, show relatively low carbon percentages, all below 1.003%. However, the olive stone bottom ash (OSBA) sample exhibited a notable carbon percentage of 6.145%. This higher carbon content may be attributed to the calcination process that the OSBA samples underwent during the experiment. During calcination at 950 °C, potassium carbonate (K_2_CO_3_) decomposes into potassium oxide (K_2_O) and carbon dioxide (CO_2_). The fact that the four raw material samples contained negligible carbon percentages and that all samples had a sulfur content of zero suggests that there was no negative impact on the geopolymerization reaction that could potentially affect the properties of the geopolymer concrete. X-ray fluorescence (XRF), determined using a Bruker M4 Tornado spectrometer (Bruker, Billerica, MA, USA), was employed to determine the chemical composition of SSCS, OSBA, CH, and SGS. The obtained values, expressed as weight percentages (wt.%), are presented in Table 3.

The slate stone contains significant amounts of silica (SiO_2_) and alumina (Al_2_O_3_), which promote an effective geopolymerization reaction in the presence of an alkaline activator solution. This observation is supported by the SSCS sample, which is primarily composed of silica (52.85 wt.%) and alumina (21.35 wt.%), yielding a SiO_2_/Al_2_O_3_ ratio of 2.47. This indicates that alkali gel formation will occur [56], contributing to the mechanical properties of the target geopolymer [57]. Other oxides, such as iron oxide (10.74 wt.%) and magnesium oxide (2.82 wt.%), were also found to play important roles in the development of suitable mechanical characteristics for the geopolymer. The calculated loss on ignition (LOI) was 5.02 wt.%.

Regarding the ceramic waste, the chamotte was predominantly composed of SiO_2_ (59.72 wt.%), Al_2_O_3_ (16.20 wt.%), and CaO (7.41 wt.%), indicating a chemical composition typical of pozzolanic activity. Its LOI was 3.45 wt.%. The OSBA was characterized by notable amounts of potassium oxide (K_2_O) (28.23 wt.%) and calcium oxide (CaO) (24.67 wt.), making it a suitable candidate for the alkaline activator in combination with commercial activators such as sodium hydroxide (NaOH), potassium hydroxide (KOH), and sodium silicate (Na_2_SiO_4_) [58]. As previously studied [23,59], CH enhances the reactivity of olive stone biomass ash, which is less reactive than fly ash [60].

Finally, the SGS composition includes several oxides, including SiO_2_ (22.54 wt.%), Al_2_O_3_ (10.81 wt.%), CaO (27.13 wt.%), and Fe_2_O_3_ (23.71 wt.%).

Comparing the elemental analysis with the chemical composition of the raw materials, it is evident that the LOI is significantly higher than the elemental carbon content. This suggests that the mass loss is not primarily due to the oxidation of organic carbon but rather to other processes, such as the decomposition of carbonates.

From the data obtained, the major components of SGS were selected for use in the phase equilibrium diagram. The main four oxides were SiO_2_, CaO, Al_2_O_3_, and Fe_2_O_3_. The components, in two groups of three oxides, were readjusted to 100% to be represented in a ternary diagram. Since the four oxides cannot be represented in the same diagram, it was decided to split the oxides in two groups of three oxides and then use the available diagrams for SiO_2_–CaO–Al_2_O_3_ and SiO_2_–CaO–Fe_2_O_3_. For each group of oxides, conversion data at 100% of the main oxides are shown for SiO_2_–CaO–Al_2_O_3_ (Table 4) and SiO_2_–CaO–Fe_2_O_3_ (Table 5).

**Figure 3:**
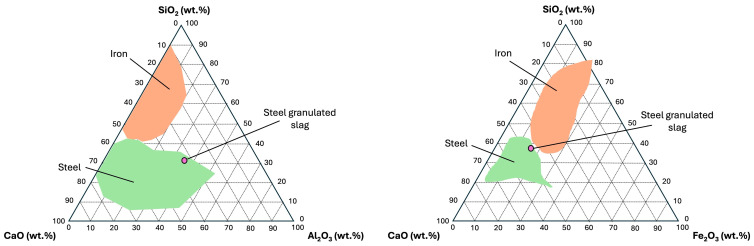
Ternary diagrams for the identification of slags based on SiO_2_, Al_2_O_3_, and CaO (**left** side) and SiO_2_, Fe_2_O_3_, and CaO (**right** side). Orange and green shaded areas represent typical areas for iron (blast furnace) and steel slag (basic oxygen furnace). Reference [64].

The Figure 3 shows both ternary diagrams representing SiO_2_–CaO–Al_2_O_3_ and SiO_2_–CaO–Fe_2_O_3_ to determine the origin of slabs based on majority of compounds (Table 3). In both ternary diagram scenarios and following the slag classification [26,61,62,63], SGS samples were more closely aligned with typical values for steel slag.

**Figure 4:**
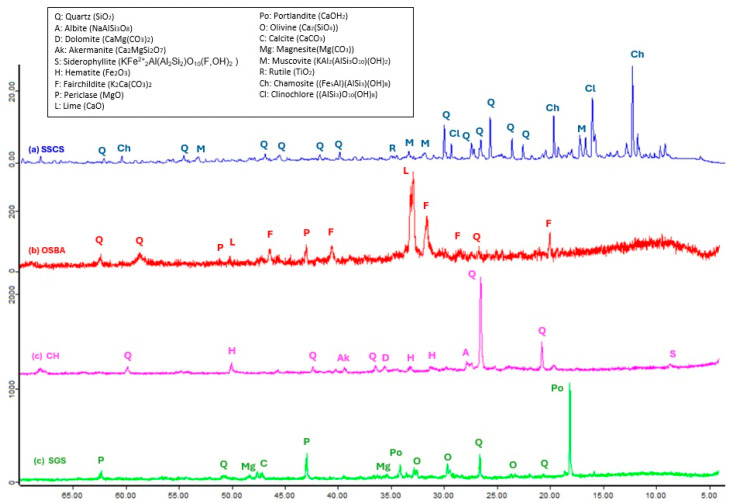
XRD patterns of SSCS, CH, OSBA, and SGS.

The chemical composition of the slate stone mining waste, SSCS, as determined by X-ray fluorescence, mainly contained silicon oxide (SiO_2_) and aluminum oxide (Al_2_O_3_). These oxides appeared in form of quartz (Q) (SiO_2_) and muscovite (M) (KAl_2_(AlSi_3_O_10_) (OH)_2_), which suggests that SSCS is a suitable source of aluminosilicates for the geopolymerization process. The presence of Fe_2_O_3_, MgO as clinochlore (Cl) ((AlSi_3_)O_10_(OH)_8_), and chamosite (Ch) ((Fe_5_Al)(AlSi_3_)(OH)_8_ was also observed in the XRF pattern. TiO_2_ was represented as rutile (R), indicative of the metamorphic nature of the mining waste. The diffraction pattern of the ceramic waste, CH, presented a certain similarity to SSCS since it showed a crystalline phase, where silicon oxide (SiO_2)_ and aluminum oxide (Al_2_O_3_) also appeared in the form of quartz (Q) (SiO_2_). Since the CH was subjected to a thermal process, Fe_2_O_3_ was represented as hematite (H) and CaO as dolomite (D) (CaMg(CO_3_)_2_) and akermanite (Ak) (Ca_2_MgSiO_7_). However, the mineralogical phases of the OSBA showed primary compounds of calcium oxide (CaO) and potassium oxide (K_2_O) in the form of fairchildite (F) (K_2_Ca(CO_3_)_2_) and siltstone (L) (CaO). The high-temperature combustion process of olive biomass creates compounds rich in carbonate such as fairchildite (F) (K_2_Ca(CO_3_)_2_). Other characteristic compounds of ash were also detected in the XRF pattern, such as siltstone (L) (CaO), traces of quartz (Q) (SiO_2_,) and periclase (P) (MgO). In the XRF pattern of the SGS, it can be seen that it is mainly composed of calcium hydroxide as portlandite (P_0_) (Ca(OH)_2_), this being a characteristic of slag related to its hydration process. This process has lower kinetics than in clinker because its dissolution is more complex due to its vitreous nature and the need for an alkaline activation source (presence of alkalis or portlandite). Other oxides such as silicon oxide, as quartz (Q)(SiO_2_), and periclase (P) (MgO) were found. Another characteristic of slag is the presence of magnesite and olivine because of they are derived from the flux via dolomite. Figure 4 shows the X-ray patterns of SSCS, OSBA, CH, and SGS. It was observed, through the identification of the crystalline phases, that the raw materials used as precursor sources and for the alkaline solution were suitable for geopolymerization, as well as exhibiting compatibility with the SGS.

The particle densities of the raw material samples were measured using the pycnometer method, as specified by the UNE-EN 1097-7:2009 standard [65]. The particle density test for the raw materials used as precursors (SSCS and CH), alkali activator solution (OSBA and Na_2_SiO_3_), and aggregates (SGS) was also carried out using the pycnometer, following the UNE-EN 1097-7:2009 standard. The calculated densities of the SSCS, OSBA, CH, and Na_2_SiO_3_ samples (2.45, 2.76, 2.53, and 2.40 g/cm^3^, respectively) were around 2.5 g/cm^3^. The density of the SGS (2.91 g/cm^3^) was slightly higher than that of the other raw materials used for the formation of the geopolymer concrete. With the calculated density values showing certain homogeneity, it can be concluded that no workability issues are foreseen during the material mixing process. For the control geopolymer, the commercial activator sodium hydroxide (NaOH) had a density of 1.39 g/cm^3^.

The pH value of each different raw material that forms the geopolymer is a key parameter for ensuring the kinetic chemical reaction process during the alkali activation of aluminosilicate sources. Therefore, the mechanical characteristics of the final geopolymer are related to the proper geopolymerization process under the correct pH alkalinity environment. Since the goal of this experimental program was to combine raw materials—(i) SSCS and CH as sources of aluminosilicates, (ii) OSBA and Na_2_SiO_3_ as the alkali activation solution, and (iii) SGS as a recycled aggregate—the geopolymer formulation must achieve a pH value that creates the right alkaline conditions for the geopolymerization process [57]. Table 6 shows the pH values of the different raw materials used in this study.

**Figure 5:**
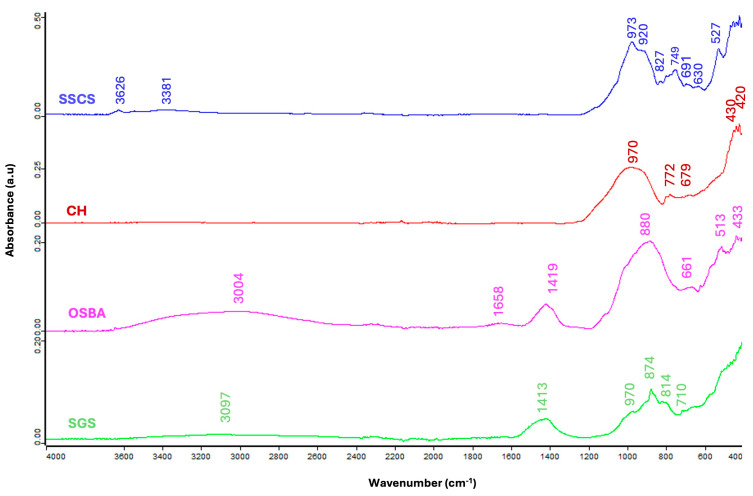
FTIR comparison of the SSCS, CH, OSBA, and SGS samples.

Attenuated total reflection Fourier transform infrared spectroscopy (ATR-FTIR) Jasco Analytica model 6800 FV (Jasco International Co., Ltd., Heckmondwike, UK) analysis was employed to identify the functional groups of SSCS, CH, OSBA, and SGS, as depicted in Figure 5.

The FTIR spectrum of the SSCS shows that bands associated with the stretching vibrations of the O-H group [33], which denote the presence of absorbed water, are located at 3381 and 3626 cm^−1^, while for the OSBA, these appear at 3004 cm^−1^. The SGS FTIR pattern shows that the stretching vibration of O-H appears at 3097 cm^−1^. The stretching vibration of the C-O group, associated with the carbonate content [19,66,67], was observed as peaks at 1419 cm^−1^ for OSBA and 1413 for SGS. Neither SSCS nor CH showed peaks related to the C-O group stretching vibration.

The bands corresponding to the stretching vibrations of Si-O-T bonds, characteristic of silica-rich materials (SiO_2_), were observed in the wavenumber range of 973 to 970 cm^−1^ for both SSCS and CH, reflecting their high silica content, as previously noted in Table 3. The bands observed around 800 cm^−1^ for OSBA, SSCS, and SGS (at 880, 827, and 874 cm^−1^, respectively) are linked to carbonate partitioning resulting from environmental exposure. The bands observed at 745, 772, and 749 cm^−1^ correspond to symmetrical Si-O-Si stretching vibrations in OSBA, CH, and SSCS, respectively.

The bands observed between 679 and 513 cm^−1^ are associated with bending vibrations resulting from the presence of quartz [66,67] in the SSCS, OSBA, and CH samples. The presence of quartz in the different raw materials was corroborated in the mineralogical composition analysis.

Finally, bands at lower wavenumbers, ranging from 527 to 420 cm^−1^, were attributed to the bending vibration of Si-O [66]. Table 7 shows the characteristic FTIR peaks for OSBA, CH, SSCS, and SGS.

### 2.5. Preparation Methodology for Control Geopolymer and Novel Geopolymer Concrete

The purpose of the applied experimental methodology was to analyze the feasibility of a novel geopolymer formed with slate stone cutting sludge and chamotte; activated by an alkali solution elaborated with olive biomass bottom ash, sodium silicate, and distilled water; and using steel slag as an aggregate.

This second stage of the research involved the physical and mechanical testing of various geopolymer families prepared with two types of geopolymer formulations for comparing the mechanical characteristics:

(i) One family of seven specimens of geopolymers, use for control purposes, was elaborated with a combination of SSCS as source of aluminosilicates and activated with an alkali activator solution composed of sodium hydroxide and sodium silicate with a Na_2_SiO_3_/NaOH weight ratio of 0.5 considering a 12 M concentration.

(ii) Four families of novel geopolymer samples were developed, each consisting of seven specimens. These were made using fixed weight percentages of SSCS and OSBA, with variations in CH content. The featured samples contain constant SSCS and OSBA weight percentages, decreasing CH from 10 to 2 wt.%, increasing SGS from 35 to 50 wt.%, and varying Na_2_SiO_3_/OSBA ratios (0.35, 0.31, 0.19, and 0.08). 

Table 8 represents the two types of formulations used in the experiment. Column 1 presents the coding used for the control geopolymer and geopolymer concrete samples. GP means geopolymer, the letter C and the digits indicate the percentage of chamotte, and the letter S followed by digits represents the percentage of steel sludge. The column labeled “NaOH Molar” indicates the molar percentage used in the control geopolymer; column 7 indicates the Na_2_SiO_3_/OSBA the weight ratio, and column 8 shows the liquid/solid weight ratio.

To compare both mixtures—the control geopolymer and novel geopolymer concrete—the different families of specimens were subjected to physical and chemical characterization. Since SSCS, CH, OSBA, and SGS were collected from outdoor landfills, prior the formation of the geopolymer, these by-products were subjected to a drying process. Samples were dried in an oven at 105 ± 2 °C for a period of 24 h to remove accumulated moisture. Once the possible moisture was removed, the next step consisted of guaranteeing the homogenization of raw materials through the selection of the finer particles. A lower particle size results in better reactivity, proper interaction between the precursor and alkali activation solution, and, therefore, higher compressive strength of the geopolymer. For the preparation of precursor raw materials, the CH was crushed and sieved to produce a powder with a particle size distribution appropriate for passing through a 0.25 mm sieve. On the contrary, the mining by-product from the slate stone cutting sludge ponds did not need grinding because of its powdery consistency from sieving. However, it was sieved to ensure a particle size of 0.25 mm.

For the preparation of the alkali activation solution, the calcined OSBA was also crushed to produce fine particles, improving its solubility in sodium silicate and distilled water. Then, the OSBA was combined with sodium silicate and distilled water according to the previously reported Na_2_SiO_3_/OSBA ratios. The slag was also kiln dried to avoid any accumulation of moisture and sieved and sorted into different particle sizes: (i) 22.4 to 20 mm, (ii) 20 to 16 mm, (iii) 16 to 10 mm, (iv) 10 to 8 mm, and 8 to 5 mm.

For the control geopolymer, the alkali solution, comprising sodium hydroxide and sodium silicate, was prepared in advance and allowed to cool for 24 h at room temperature to mitigate the exothermic reaction, prior to being combined with SSCS in the proportions specified in Table 8.

Once the precursor materials were prepared and the two different alkali activators were elaborated, the subsequent step was to weigh the precursor materials, alkali solution, and steel granulated slag for the novel geopolymer. The control geopolymer pastes were poured into prismatic molds with internal dimensions of 35 × 35 × 35 mm, producing seven specimens for each formulation. The novel geopolymer concrete pastes were set in prismatic molds of 100 mm × 100 mm × 100 mm for the seven specimens. Samples of both formulations were vibrated on a vibration table for 30 s.

The curing process of the geopolymer samples followed a three-stage cycle over 28 days: (i) curing at ambient temperature (25 °C) for 24 h, (ii) then placing the samples in an oven at 85 ± 5 °C for 90 h, and (iii) finally curing them at ambient temperature until day 28 for the completion of the cycle.

**Figure 6:**
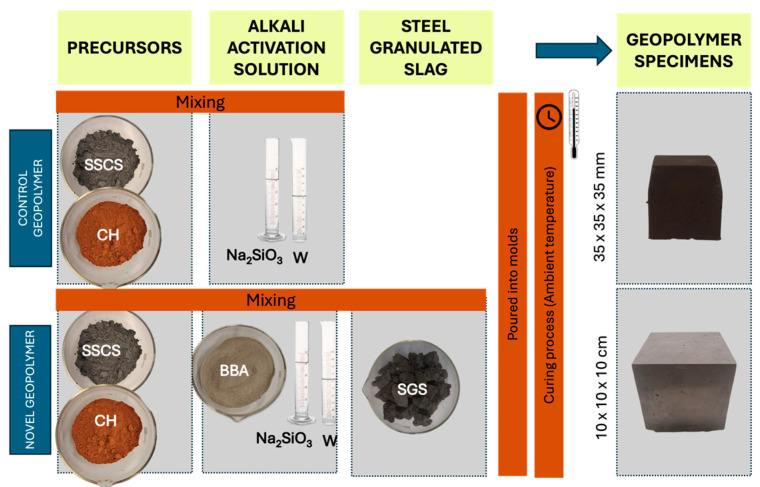
Schematic process of geopolymer preparation with SSCS, CH, OSBA, Na_2_SiO_3_, and SGS. (W: distilled water).

Figure 6 provides a schematic overview of the process.

The two-stage curing process was designed to enhance the physical and mechanical properties of the geopolymers by influencing the polymerization reaction and microstructure development.

In the first stage, curing at room temperature promotes initial gelation, which is crucial for forming the geopolymer’s three-dimensional structure. During this stage, dissolved silicon and aluminum species begin reacting to form an amorphous aluminosilicate gel. This phase is vital for ensuring an even distribution of reaction products and preventing crack formation due to sudden contractions.

The second stage, conducted at 85 °C, accelerates the polymerization reaction and fosters the development of a more crystalline microstructure. The elevated temperature enhances ion mobility, leading to greater densification of the matrix. This heat treatment also improves the material’s mechanical strength and durability by eliminating non-structural water and consolidating the geopolymer’s internal structure.

Finally, controlled cooling stabilizes the microstructure formed during the thermal treatment, preventing internal stresses or cracks that might result from rapid temperature fluctuations.

After completing the second stage of the curing process (90 h at 85 ± 5 °C) in the three-stage procedure, all specimens were carefully demolded and stored under controlled laboratory conditions to ensure consistency throughout the testing period. The geopolymer concrete samples were then allowed to cool gradually to room temperature, after which they were stored in sealed, labeled plastic bags at approximately 23 ± 5 °C to complete the 28-day curing process. The use of sealed plastic bags helped minimize moisture loss and prevent carbonation.

Tests were conducted at 24 h intervals, except for the compressive strength tests performed at 7, 14, and 28 days of curing. During the periods between tests, the geopolymer samples remained sealed in the labeled bags to maintain a stable internal environment and prevent external interference. Additionally, one sample from each geopolymer family was set aside and stored under the same conditions for subsequent analysis using scanning electron microscopy (SEM). (Microscope Carl Zeiss Merlin, Zeiss GmbH, Jena, Germany).

Physical and mechanical tests were carried out to identify phenomena such as linear shrinkage and weight loss, which can occur during the geopolymerization process. Changes in weight and dimensions after curing serve as critical indicators for construction materials and are important considerations in the sustainable reuse of various by-products. Additionally, the effect of water on the novel geopolymer samples was assessed through capillary water absorption and cold-water absorption tests. These standardized tests measured the samples’ ability to absorb water and, consequently, their mass variation. To evaluate the mechanical properties of the geopolymer samples, bulk density, open porosity, and compressive strength tests were conducted for comparison with other construction materials. The methodology applied in this experimental program adhered to the following standards: (i) mass loss (UNE-EN 13581:2003 Standard) [68]; (ii) linear shrinkage (UNE-EN 13872:2004 Standard) [69]; (iii) capillary water absorption (UNE-EN 1015-18:2003 Standard) [70]; (iv) bulk density and open porosity (UNE-EN 1015-10:2000 Standard) [71]; and (v) compressive strength (UNE-EN 1015-10:2020 Standard) [72]. Finally, the geopolymer samples were subjected to Fourier transform infrared spectroscopy (FTIR) and scanning electron microscopy (SEM).

## 3. Results

### 3.1. Determination of Mass Loss

**Figure 7:**
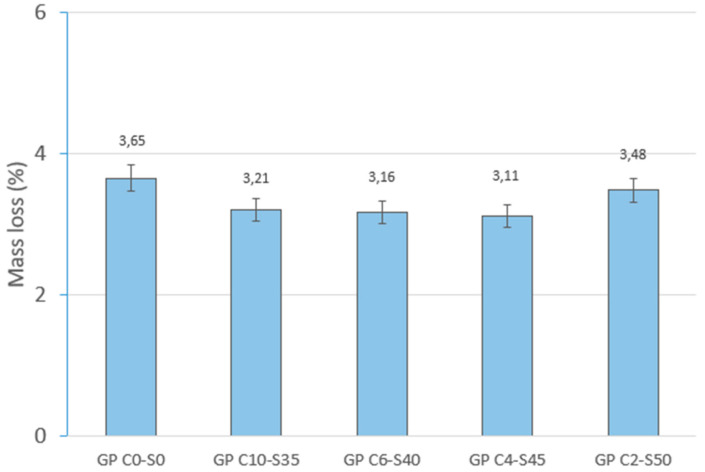
Mass loss of control geopolymer and novel geopolymer concrete using SSCS, CH, OSBA, Na_2_SiO_3_, and SGS.

The percentage of mass loss is related to the loss of free water in the mortar. The control family (GP C0-S0) was formed with 8% H_2_O, while in the rest of the families studied. the percentage of water remained constant (18%). GP C0-S0 showed a lower mass loss (3.65%) than GP C10-S35 (3.21%) and GP C6-S40 (3.16%) during the curing process. However, when aggregates were incorporated into the mix, the mass loss was reduced, reaching its lowest value (3.11%) when the aggregate content was 45% (GP C4-S45). This can be attributed to the fact that the SGS improves the efficiency of the geopolymer reaction by providing a larger interaction surface area and contributing to the retention of water in the pores and on the surface of the particles. However, as the aggregate content increased to 50% (GP C2-S50), the mass loss showed an increase (3.48%), suggesting that an excess of SGS may affect the cohesion of the geopolymer matrix and facilitate the release of entrapped water. Figure 7 shows the mass loss percentages (%) of the geopolymer families.

### 3.2. Determination of Linear Shrinkage

**Figure 8:**
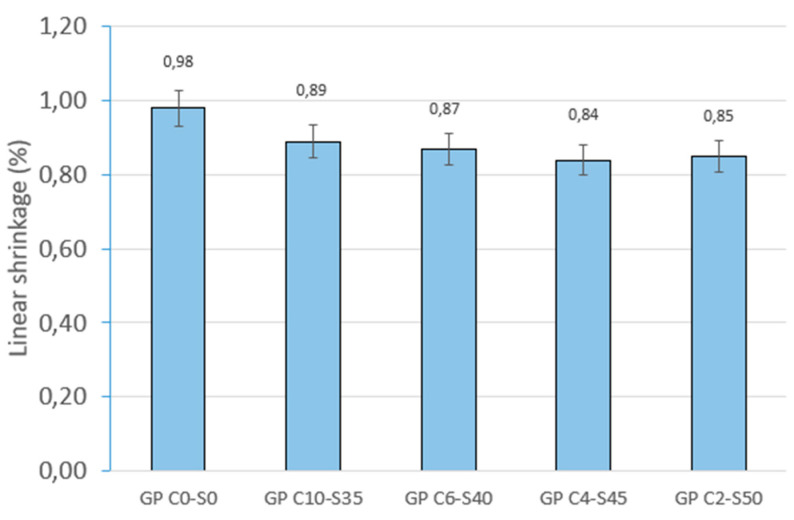
Linear shrinkage of control geopolymer and novel geopolymer concrete using SSCS, CH, OSBA, Na_2_SiO_3_, and SGS.

The results obtained for linear shrinkage (%) (Figure 8) show that GP C0-S0 presents the highest shrinkage (0.98%). As SGS is incorporated into the mix, the shrinkage decreases progressively, reaching its lowest value in GP C4-S45 (0.84%). However, in the sample with the highest aggregate content (GP C2-S50), shrinkage exhibits a slight increase (0.85%).

These results are consistent with the trend observed for mass loss. The presence of SGS reduces geopolymer shrinkage by restricting matrix movement and decreasing the amount of free water available for evaporation. SGS acts as a structural support that limits the shrinkage of the material during curing. However, when the SGS content reaches 50%, a lack of cohesion with the geopolymer mortar is observed. Therefore, there is a slight reduction in the efficiency of the chemical reaction and a slight increase in shrinkage.

### 3.3. Capillary Water Absorption and Cold-Water Absorption Determination

**Figure 9:**
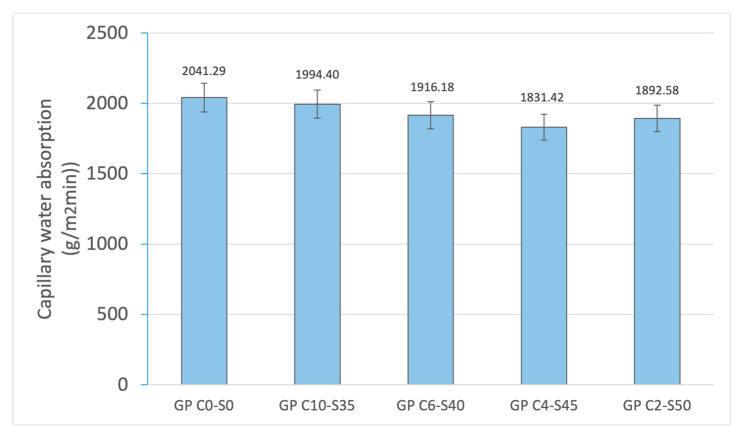
Capillary water absorption of control geopolymer and novel geopolymer concrete using SSCS, CH, OSBA, Na_2_SiO_3_, and SGS.

**Figure 10:**
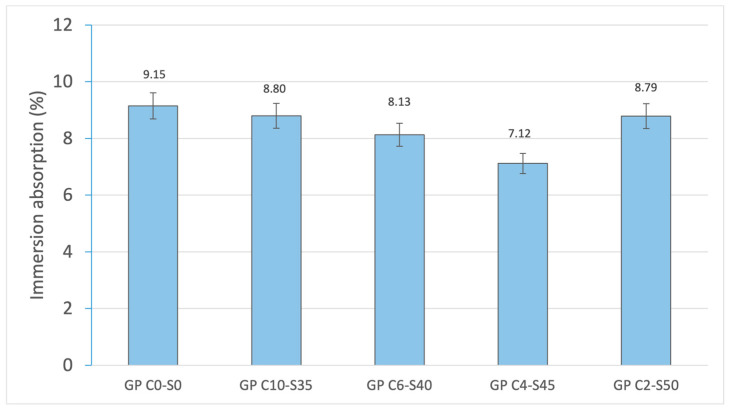
Cold-water absorption of control geopolymer and novel geopolymer concrete using SSCS, CH, OSBA, Na_2_SiO_3_, and SGS.

The results of water absorption by capillary action (Figure 9) and by immersion (Figure 10) show the behavior of the geopolymer matrix with respect to water absorption, providing information on its porosity and internal structure. The control geopolymer (GP C0-S0) presents the highest values in both measures, with 2041.29 g/m^2^-min in capillary absorption and 9.15% in immersion absorption. This suggests a more porous structure with a greater number of interconnected pores, which facilitates the entry and mobility of water within the material. As SGS is incorporated, water absorption progressively decreases, reaching its lowest point in GP C4-S45 for capillary absorption (1831.42 g/m^2^-min) and immersion absorption (7.12%). This indicates that the presence of SGS improves the compaction of the geopolymer matrix, reducing pore connectivity and limiting the amount of water that the material can absorb by both capillary and immersion absorption. However, when the SGS content reaches 50% (GP C2-S50), a slight increase in both properties are observed (1892.58 g/m^2^-min for capillary absorption and 8.79% for immersion absorption). This behavior suggests that an excessive proportion of aggregate may affect the cohesion of the geopolymer matrix, generating a less uniform distribution of pores or even microcracks, which again favors water absorption. The observed trend indicates that the incorporation of SGS reduces water absorption, which is related to the reduced number of connected pores in the geopolymer matrix, as SGS fills spaces and restricts moisture absorption. This trend is consistent with decreased linear shrinkage and mass loss.

### 3.4. Determination of Open Porosity

**Figure 11:**
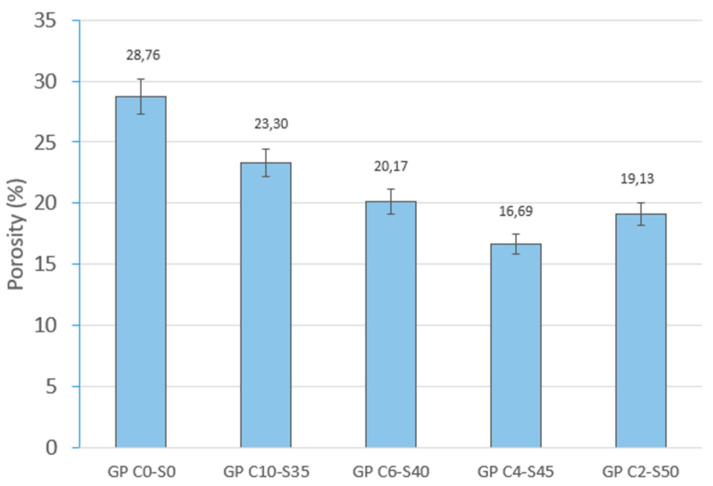
Open porosity of control geopolymer and novel geopolymer concrete using SSCS, CH, OSBA, Na_2_SiO_3_, and SGS.

GP C0-S0 has the highest porosity (28.76%), which explains its higher water absorption by capillary absorption (2041.29 g/m^2^-min) and immersion (9.15%). The absence of SGS generates a matrix with a more open structure, facilitating water mobility within the material. When SGS is incorporated, porosity decreases progressively, reaching its lowest value in GP C4-S45 (16.69%). This reduction in porosity is related to the lower water absorption by capillary absorption (1831.42 g/m^2^-min) and immersion (7.12%). This suggests that an adequate proportion of SGS contributes to the density of the geopolymeric matrix, decreasing voids and improving the dimensional stability of the material. Increasing the SGS content to 50% in the geopolymeric matrix produces an increase in porosity (18.13%) related to the presence of microcracks due to the lack of geopolymeric mortar. Figure 11 shows the porosity results of the five families studied.

### 3.5. Determination of Bulk Density

**Figure 12:**
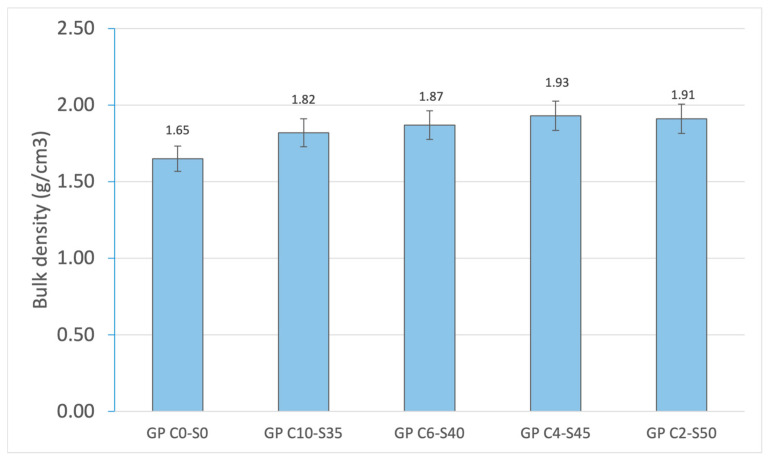
Bulk density of control geopolymer and novel geopolymer concrete using SSCS, CH, OSBA, Na_2_SiO_3_, and SGS.

The bulk density allows for an evaluation of the degree of compaction of the geopolymer matrix. The presence of OSBA in the matrices of GP C10-S35, GP C6-S40, GP C4-S45, and GP C2-S50 produces a higher degree of densification related to the increase in Ca present. Therefore, GP C0-S0 presents the lowest density (1.65 g/cm^3^), confirming its more porous structure and higher water absorption. As SGS is incorporated, the density increases progressively, reaching its maximum at GP C4-S45 (1.93 g/cm^3^). This increase in density is associated with reduced porosity (16.69%) and a higher SGS content (45%). The slight decrease in density in GP C2-S50 (1.91 g/cm^3^) is related to the structural problems suffered by the specimen related to the cracks produced because of the lack of binder. Figure 12 shows the bulk density results obtained from the experiment.

### 3.6. Compression Strength Test Results

**Figure 13:**
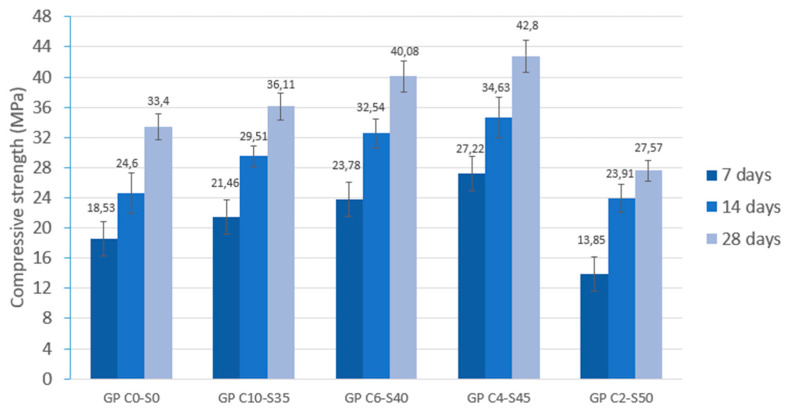
Compressive strength of control geopolymer and novel geopolymer concrete using SSCS, CH, OSBA, Na_2_SiO_3_, and SGS.

The compressive strength of the geopolymers was determined at 7, 14, and 28 days of curing. The curing conditions were the same for all the series of geopolymers produced, so the compressive strength values depended directly on the reactivity of the blends. Figure 13 shows the compressive strength results obtained at 7, 14, and 28 days of curing. It is observed that the curing time of the specimens influences the compressive strength values with a gradual increase during the curing process. The increase intensifies between 13 and 28 days of curing, which is associated with the repolymerization process that occurs during gel formation as a result of alkaline activation. GP C0-S0 showed a compressive strength of 33.4 MPa, which was related to the lack of SGS in its composition to serve as an aggregate. GP C4-S45, with 45% SGS, showed the best compressive strength results (42.8 MPa) at 28 days, related to its optimal binder/SGS ratio. There was a decrease in the strength values of the GP C2-S50 specimen due to microcracks on the surface caused by the lack of binder.

### 3.7. Scanning Electron Microscopy (SEM) and Energy-Dispersive X-Ray Spectroscopy (EDX) Analysis of Control Geopolymers and Novel Geopolymers

**Figure 14:**
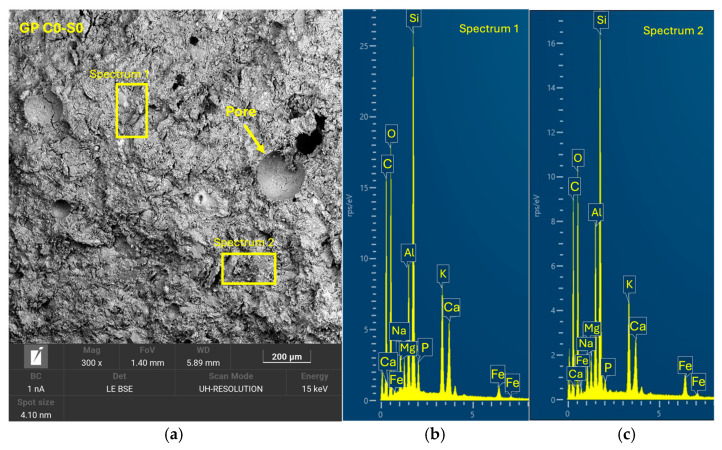
SEM results for the control geopolymer sample GP C0-S0: (**a**) entire area of the secondary SEM 3000× image with the selection of two points for spectral analysis; (**b**) EDX analysis of spectrum 1; (**c**) EDX analysis of spectrum 2.

**Figure 15:**
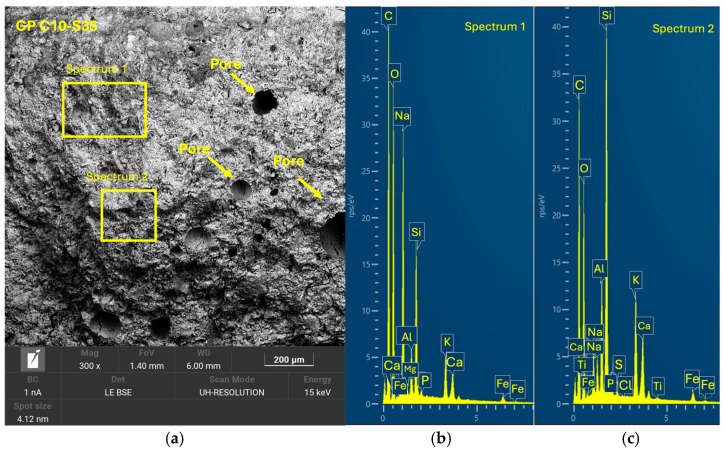
SEM results for the novel geopolymer sample GP C10-S35: (**a**) entire area of the secondary SEM 3000× image with the selection of two points for spectral analysis; (**b**) EDX analysis of spectrum 1; (**c**) EDX analysis of spectrum 2.

**Figure 16:**
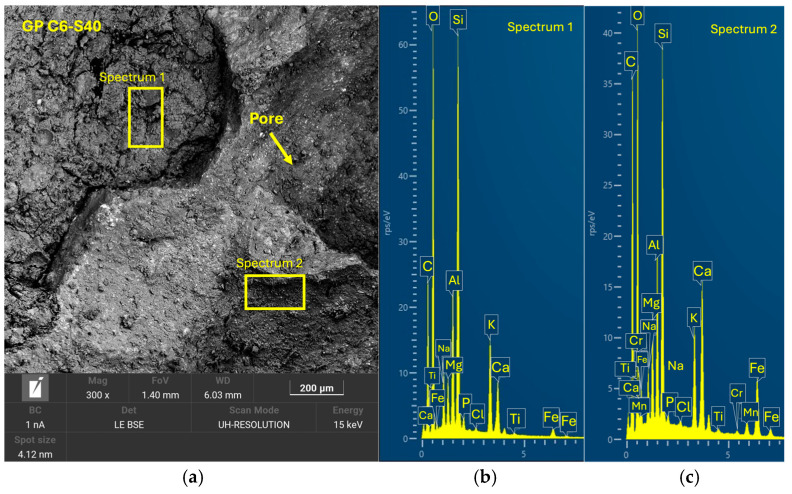
SEM results for the novel geopolymer sample GP C6-S40: (**a**) entire area of the secondary SEM 3000× image with the selection of two points for spectral analysis; (**b**) EDX analysis of spectrum 1; (**c**) EDX analysis of spectrum 2.

**Figure 17:**
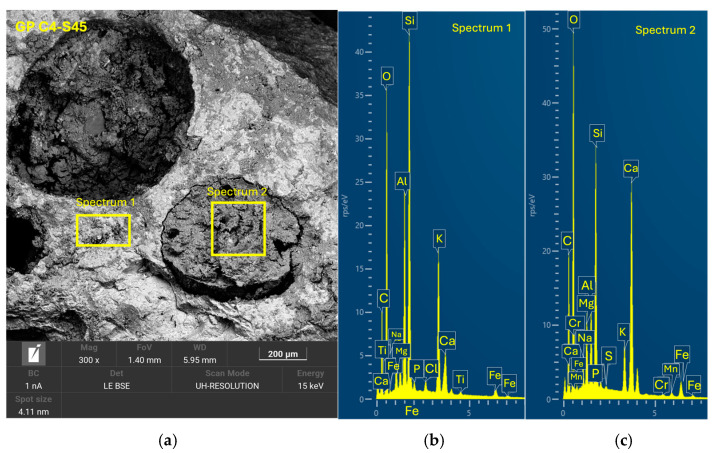
SEM results for the novel geopolymer sample GP C4-S45: (**a**) entire area of the secondary SEM 3000× image with the selection of two points for spectral analysis; (**b**) EDX analysis of spectrum 1; (**c**) EDX analysis of spectrum 2.

After 28 days of curing, the five geopolymer families were analyzed by SEM-EDX to evaluate the microstructure and reaction efficacy. GP C0-S0 (Figure 14), used as a reference without SGS incorporation, showed a compact structure with a well-consolidated homogeneous matrix. It exhibited the presence of reaction products with a uniform distribution and a morphology characterized by the absence of significant cracks. Figure 15 shows that sample GP C10-S35 presented a homogeneous and compact structure with a lower amount of SGS incrustations, leading to a lower compressive strength. In the case of GP C6-S40 (Figure 16) and GP C4-S45 (Figure 17), the incorporation of SGS in percentages of 40% and 45%, respectively, favored the formation of a dense structure with a low pore volume. In the EDX analyses, phases rich in Ca, Na, Si, Al, and K were identified, confirming the presence of (N, C)-A-S-H gels. The higher proportion of SGS in these formulations promoted a more uniform and cohesive matrix, which may be asso-ciated with higher mechanical strength compared to other families. The reduction in the Si/Al molar ratio in these formulations favored the formation of a dense and homogeneous geopolymeric network, which reinforces the hypothesis that an adequate proportion of SGS contributes to improve matrix cohesion.

**Figure 18:**
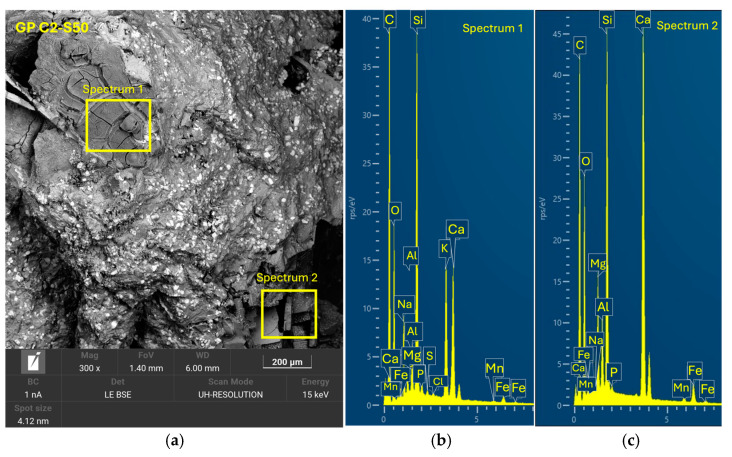
SEM results for the novel geopolymer sample GP C2-S50: (**a**) entire area of the secondary SEM 3000× image with the selection of two points for spectral analysis; (**b**) EDX analysis of spectrum 1; (**c**) EDX analysis of spectrum 2.

On the other hand, the GP C2-S50 family (Figure 18), with the highest SGS content (50%), presented a more dispersed structure with higher porosity, indicating that the excess SGS and the lower amount of precursor material reduced the efficiency of the geopolymeri-zation reaction. In this formulation, less compact regions with less adherence between phases were observed, suggesting that a high amount of slag may affect the continuity of the geopolymeric matrix. Another relevant aspect is the influence of Na₂SiO₃ content on the workability and cohesion of the mixture. In formulations with lower Na₂SiO₃ content, such as GP C2-S50, a lower adhesion between components was observed, which affected the compactness of the system and, consequently, decreased the mechanical strength. This is because lower Na₂SiO₃ content reduces the availability of soluble Si species for the formation of (N, C)-A-S-H gels, which negatively impacts structural integrity.

## 4. Conclusions

After performing the physicochemical characterization of the four different raw materials, as well as physical and mechanical testing of the various geopolymer families, a set of conclusions was drawn. The primary goal of this study was to assess the viability of combining by-products from the mining, ceramics, olive production, and steel manufacturing industries as a sustainable geopolymer concrete.

The key conclusions derived from this study are as follows:

- The elemental analysis of the studied raw materials revealed negligible levels of carbon, hydrogen, and nitrogen. As a result, it was anticipated that the geopolymerization process would not be affected or compromised.
- Raw materials as precursors: The chemical composition analysis of the SSCS revealed significant amounts of silica (52.85 wt.%) and alumina (21.35 wt.%). The XRF analysis of the CH indicated pozzolanic activity, primarily due to its high silica content (59.72 wt.%) and low alumina content (16.20 wt.%). The combination of these two raw materials, with their specific silica and alumina contents, offered a potential source of aluminosilicates for the experiment
- Raw material as a component of the alkali activation solution: The chemical composition analysis of the olive stone biomass bottom ash revealed that it was primarily composed of K_2_O and CaO, with respective percentages of 28.23 wt.% and 24.67 wt.%.
- Raw material as a recycled aggregate: The SGS composition analysis revealed oxides such as silica (SiO_2_), alumina (Al_2_O_3_), calcium oxide (CaO), and iron oxide (Fe_2_O_3_), with corresponding weight percentages of 22.54%, 10.81%, 27.13%, and 23.71%, respectively.
- The optimal novel geopolymer concrete formula, GP C4-S45, was created with 20.0 wt.% of SSCS, 4.0 wt.% of CH, 45.0 wt.% of SGS, Na_2_SiO_3_/OSBA ratios of 0.19, and constant amount of OSBA (8 wt.%). The maximum obtained compressive strength was 42.8 MPa at 28 days of curing.
- It was observed that increasing the amount of SGS up to 45 wt.% and reducing the amount of CH to 4 wt.% increased the degree of reactivity of the geopolymer. The chemical composition of SGS, which is high in CaO (27.13 wt.%), enhances reactivity in the geopolymerization process when incorporated into the formulation, leading to a reduction in the amount of CH. The formula’s equilibrium shows a higher concentration of CaO derived from the slag, which helps to eliminate the excess CaO present in CH. Furthermore, the Fe_2_O_3_ (23.71 wt.%) content in SGS contributes to its excellent mechanical properties, resulting in significant compressive strength, as demonstrated in initial investigations using these by-products.
- The higher proportion of SGS in the formulation fostered a more uniform and cohesive matrix, which was likely linked to the increased mechanical strength compared to other formulations. The reduction in the Si/Al molar ratio in these mixtures facilitated the formation of a dense and homogeneous geopolymeric network, supporting the hypothesis that an optimal SGS proportion enhances matrix cohesion.
- The physical, chemical, mechanical, and microstructural characterization results will support the process’s adaptation for industrial-scale applications. To scale up production, the materials can be organized in hoppers and mixed in industrial mixers, with the alkaline activator prepared separately in another mixer. The process’s low curing temperature requirements make it well suited for efficient scalability in industrial settings. However, is recommended to study the feasibility of this geopolymer concrete with steel bars.

The results show that the presence of steel slag as an aggregate in the geopolymer had a positive effect on the promotion of a circular economy in the construction sector, saving energy, reducing carbon emissions in the manufacturing of this type of construction material, and providing a solution for the uncontrolled landfill deposition of the studied by-products.

## Figures and Tables

**Table 1:** Particle size distribution of SSCS, OSBA, and CH.

Distribution (μm)	D10	D50	D90
SSCS ^1^	1.602	5.179	11.991
OSBA ^2^	17.580	70.618	51.404
CH ^3^	3.354	51.404	239.542

^1^ Slate stone cutting sludge; ^2^ olive stone bottom ash; ^3^ chamotte.

**Table 2:** Elemental analysis of SSCS, OSBA, CH, and SGS.

Sample	Nitrogen, %	Carbon, %	Hydrogen, %
SSCS ^1^	0.104 ± 0.003	1.003 ± 0.057	0.315 ± 0.002
OSBA ^2^	0.101 ± 0.003	6.145 ± 0.057	2.660 ± 0.002
CH ^3^	0.001 ± 0.003	0.423 ± 0.057	0.014 ± 0.002
SGS ^4^	0.000 ± 0.003	0.042 ± 0.057	0.080 ± 0.002

^1^ Slate stone cutting sludge; ^2^ olive stone bottom ash; ^3^ chamotte; ^4^ steel granulated slag.

**Table 3:** X-ray fluorescence of SSCS, OSBA, CH, and SGS.

Compound	SSCS, wt.%	OSBA, wt.%	CH, wt.%	SGS, wt.%
SiO_2_	52.85	4.22	59.72	22.54
Al_2_O_3_	21.35	0.670	16.20	10.81
CaO	0.402	24.67	7.41	27.13
Fe_2_O_3_	10.74	0.91	7.40	23.71
K_2_O	4.22	28.23	5.10	0.029
MgO	2.82	3.82	2.43	7.14
TiO_2_	1.27	0.058	0.86	0.684
Na_2_O	1.06	0.759	0.41	0.284
SO_3_	0.425	0.120	0.81	0.181
P_2_O_5_	0.23	1.81	0.18	0.259
LOI ^1^	5.02	20.15	3.45	-

^1^ Loss on ignition.

**Table 4:** Recalculated data at 100% for the ternary diagram of SiO_2_–CaO–Al_2_O_3._

Oxide	XFR Data, wt.%	Recalculation at 100 wt.%
SiO_2_	22.54	37.26
CaO	27.13	44.86
Al_2_O_3_	10.81	17.88

**Table 5:** Recalculated data at 100% for the ternary diagram of SiO_2_–CaO–Fe_2_O_3._

Oxide	XFR Data, wt.%	Recalculation at 100 wt.%
SiO_2_	22.54	30.72
CaO	27.13	36.98
Fe_2_O_3_	23.71	32.30

**Table 6:** pH determination of SSCS, OSBA, CH, SGS, NaOH, and Na_2_SiO_3_.

Raw Material	pH Average	Temp. (°C)
SSCS ^1^	8.58	24.8
OSBA ^2^	11.75	24.5
CH ^3^	8.66	24.4
SGS ^4^	12.35	24.4
NaOH ^5^	13.7	24.5
Na_2_SiO_3_^6^	13.0	24.6

^1^ Slate stone cutting sludge; ^2^ olive stone bottom ash; ^3^ chamotte; ^4^ steel granulated slag; ^5^ sodium hydroxide; ^6^ sodium silicate.

**Table 7:** Characteristic absorption peaks of the FTIR spectra for the OSBA, CH, and SSCS samples.

Function Group	Wavenumber Range (cm^−1^)					FTIR Peaks (cm^−1^)
Raw Material		OSBA	CH	SSCS	SGS	Reference
Stretching vibration O-H	3626–3004	3004	-	3626, 3381	3097	[33]
Asymmetric stretching vibration C-O	1419–1413	1419	-	-	1413	[19,66,67]
Asymmetric stretching vibration Si-O-T	973–970	-	970	973	-	[66,67]
C-O bond vibrations in carbonate groups	880–827	880	-	827	874	[66,67]
Bending symmetric stretching vibration Si-O-Si	772–745	745	772	749	-	[66,67]
Bending vibration in quartz	679–513	661, 513	679	630	-	[66]
Bending vibration Si-O	527–420	513, 433	430, 420	527, 427	431	[66]

**Table 8:** Relationship of the geopolymer families of the control geopolymer and novel geopolymer samples with SSCS, CH, OSBA, Na_2_SiO_3_, and SGS.

Sample Series Code	SSCS (%)	CH (%)	NaOH Molar (%)	OSBA (%)	SGS (%)	Na_2_SiO_3_/OSBA Weight Ratio	Liquid/SolidWeight Ratio
GP C0-S0	70.0	0	12	-	-	-	0.43
GP C10-S35	20.0	10.0	-	8.0	35.0	0.35	1.17
GP C6-S40	20.0	6.0	-	8.0	40.0	0.31	1.93
GP C4-S45	20.0	4.0	-	8.0	45.0	0.19	1.95
GP C2-S50	20.0	2.0	-	8.0	50.0	0.08	1.27

## Data Availability

The original contributions presented in this study are included in the article. Further inquiries can be directed to the corresponding author.
